# Tracking effects of extreme drought on coniferous forests from space using dynamic habitat indices

**DOI:** 10.1016/j.heliyon.2024.e27864

**Published:** 2024-03-20

**Authors:** Mojdeh Safaei, Till Kleinebecker, Manuel Weis, André Große-Stoltenberg

**Affiliations:** a Division of Landscape Ecology and Landscape Planning, Institute of Landscape Ecology and Resource Management, IFZ Research Centre for Biosystems, Land Use and Nutrition, Justus Liebig University Giessen, Heinrich-Buff Ring 26-32, 35392, Giessen, Germany; b Center for International Development and Environmental Research (ZEU), Senckenbergstrasse 3, 35390, Giessen, Germany; c Hessian Agency for Nature Conservation, Environment and Geology (HLNUG), Rheingaustraße 186, 65203, Wiesbaden, Germany

**Keywords:** Dynamic habitat indices, Coniferous forest, Ecosystem health, Extreme drought events, Germany

## Abstract

Terrestrial ecosystems such as coniferous forests in Central Europe are experiencing changes in health status following extreme droughts compounding with severe heat waves. The increasing temporal resolution and spatial coverage of earth observation data offer new opportunities to assess these dynamics. Dense time-series of optical satellite data allow for computing Dynamic Habitat Indices (DHIs), which have been predominantly used in biodiversity studies. However, DHIs cover three aspects of vegetation changes that could be affected by drought: annual productivity, minimum cover, and seasonality. Here, we evaluate the health status of coniferous forests in the federal state of Hesse in Germany over the period 2017–2020 including the severe drought year of 2018 using DHIs based on the Normalized Difference Vegetation Index (NDVI) for drought assessment. To identify the most important variables affecting coniferous forest die-off, a series of environmental variables together with the three DHIs components were used in a logistic regression (LR) model. Each DHI component changed significantly across non-damaged and damaged sites in all years (*p*-value 0.05). When comparing 2017 to 2019, DHI-based annual productivity decreased and seasonality increased. Most importantly, none of the DHI components had reached pre-drought conditions, which likely indicates a change in ecosystem functioning. We also identified spatially explicit areas highly affected by drought. The LR model revealed that in addition to common environmental parameters related to temperature, precipitation, and elevation, DHI components were the most important factors explaining the health status. Our analysis demonstrates the potential of DHIs to capture the effect of drought events on Central European coniferous forest ecosystems. Since the spaceborne data are available at the global level, this approach can be applied to track the dynamics of ecosystem conditions in other regions, at larger spatial scales, and for other Land Use/Land Cover types.

## Graphical abstract

**Figure:**
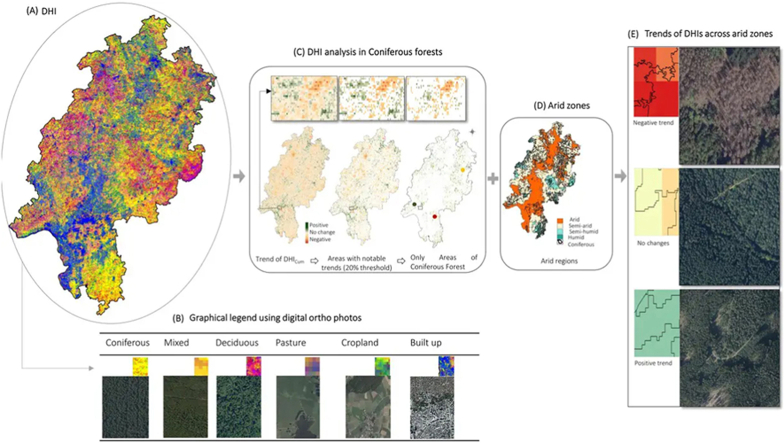

## 1 Introduction

Severe drought events and heatwaves currently represent major driving forces behind forest die-off [[1], [2], [3]], and the risk of megadrought events is likely to increase [4]. This can push forest ecosystems beyond their historic range of disturbance [5] and thereby posing a threat to forest resilience [3,[6], [7], [8], [9]] and can cause vast changes in vegetation dynamics [[10], [11], [12], [13]]. Forests in Germany, as in other central European countries, have encountered droughts and heatwaves of various magnitudes in recent years [9]. The extreme droughts between 2018 (with 3.3 °C higher than the long-term average from 1961 to 1990 [14]) and 2020 severely affected Central Germany, where in certain parts in central Germany up to two-thirds of coniferous forests died [15] and certain tree species such as Spruce Scots pine (*Pinus sylvestris* L.) potentially having reached tipping point [16].

Remote sensing of vegetation indices enables the monitoring of drought conditions at large spatial extents and could support monitoring and management efforts accounting for the dynamic nature of ecosystem health [7,17,18]. For example, continuous space-borne measures such as phenological metrics [19] provide more direct links to ecosystem functioning than discrete classifications [20]. Consequently, metrics calculated from remotely sensed time series data such as Dynamic Habitat Indices (DHIs), which rely on productivity properties, such as vegetation greenness or degree of vegetation seasonality, provide a baseline of the (natural) variability in ecosystem health and degradation [21,22].

DHIs are useful tools to summarize measures of vegetative productivity including the cumulative and minimum annual productivity as well as variation in annual productivity [21]. DHIs have been derived at 1 km spatial resolution for Australia [23], Canada (Coops et al., 2008), and recently at global scale [24]. The availability of remote sensing products such as the Normalized Difference Vegetation Index (NDVI), Leaf Area Index (LAI), the fraction of light absorbed by the vegetation (fPAR), or estimates of Gross Primary Productivity (GPP) derived from globally available MODIS satellite data clearly facilitates the calculation of DHIs [21,25]. However, with the increasing availability of remote sensing products with high temporal resolution, for example from the European Copernicus Land Monitoring service that provides global NDVI data at 300 m resolution with an interval of 10 days [26], these high temporal scale data products have a great potential for exploring ecosystem dynamics using composite vegetation indices such as the DHI.

The increasing temporal extent of baseline data to compute DHIs also allows for analyzing changes in DHIs over successive years across Land Use Land Cover types [27]. For example, identifying significant deviations from the long-term mean or a baseline state can facilitate to demarcate regions undergoing changes in ecosystem conditions [21,25,28,29]. To the best of our knowledge, DHIs have not been used to explore the health characteristics of coniferous forests affected by drought, yet. This is even more remarkable as DHIs may hold a substantial potential to refine the understanding between vegetation dynamics [24,28] and severe drought events [15].

Here, we present a detailed spatiotemporal analysis of a NDVI-based DHI across coniferous forests of Hesse, Germany. The specific objectives include (1) establishing a Hessian dynamic habitat index (DHI_Total_) using spaceborne NDVI data at 300 m resolution, (2) identifying the DHI based health status of the coniferous forest before the severe drought event in 2018 and its potential recovery afterwards, (3) identifying where the drought event had strongest effects using spatiotemporal analysis, and (4) exploring the relationship between the environmental drivers, DHI components and coniferous health status.

## 2 Material and methods

### 2.1 Study area

**Fig. 1:**
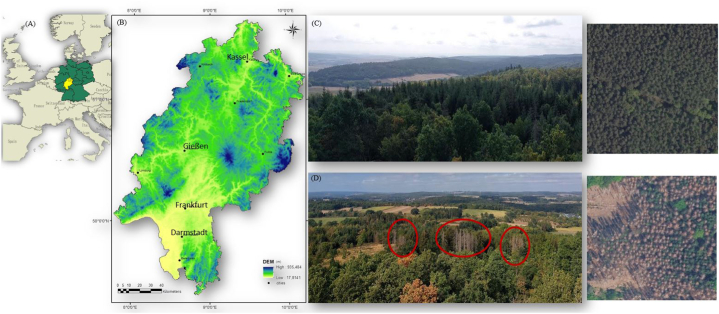
(A) Location of the federal state of Hesse in central Germany and in Europe (B) the digital elevation model (DEM) [33] ranging between 75 and 950 m above sea level. Photos show some indication of the health status of coniferous forests: (C) example of a non-damaged site and (D) of a damaged site. Photos were taken by the first-author in August 2022 in the Vogelsberg region and aerial overview pictures are derived from digital orthophotos of the Hessian geoportal [34].

This study was conducted in the federal state of Hesse, Germany (Fig. 1). The mean annual temperature (1981–2010) is 8.8 °C. It increased from 8.2 °C between 1951 and 1980, and it is still rising. There is a considerable spatial variation of annual increase ranging from almost 0 in some northern parts to +1.4 °C in the south. The annual precipitation increased slightly from 735 mm (1901–1930) to 807 mm (1981–2010). As for precipitation, there is a clear spatial variability of precipitation ranging from around 500 mm in the Upper Rhine Valley to 1400 mm at the higher elevations of e.g. the Vogelsberg or the Rhön [30].

According to the Climate Protection Scenario in Hesse, an increase in the number of particularly hot days with temperatures above 30 °C is very likely [30]. Precipitation is predicted to shift from summer to winter and will be more likely fall as rain instead of snow in winter. The probability and the severity of heavy precipitation and drought events will increase in the future [31].

In Hesse, forests cover more than 40 % of the land surface (about 894,000 ha). More than half of these are dominated by broad-leaved tree species such as *Fagus sylvatica* L. (Beech) and *Quercus robur* L (Oak). Beech and oak together account for 43.8% of the forest area. Conifers make up almost 40%. The main coniferous tree species are *Picea abies* (L.) Karst. (Spruce)*, Pinus sylvestris* L. (Scots pine), *Pseudotsuga menziesii* Franco (Douglas fir)*,* and *Abies alba* Mill. (silver fir) [32]. As a result of storm events (and subsequent bark beetle infestation), the area of spruce forests decreased by more than 20,000 ha between 2002 and 2012. This was associated with an increase in deciduous forests and Douglas fir, which is more and more used as a “replacement” for spruce [32].

### 2.2 Data

#### 2.2.1 Satellite data

To compute the DHI_Total_, we used the NDVI product of the Copernicus Global Land Service with a spatial resolution of 300 m [26]. It is a 10-daily synthesis product derived from PROBA-V observations [26]. We used data from Jan 01, 2017 until Dec 31, 2020, that showed consistent data value ranges, to calculate the Hessian DHIs to examine the sudden effects of the extreme drought of 2018. Recent studies demonstrated the applicability of the DHI for similar temporal ranges [[35], [36], [37]]. Further, recent analysis of annual, satellite-derived NDVI data showed the effect of the 2018 drought on ecosystem conditions in Europe [38] including central European forests [14,39]. Notably, such data can potentially be applied to track coniferous forest health [16]. In addition to NDVI data, a satellite-based digital elevation model (DEM) [33] was used to derive elevation above sea level as an topographical predictor of the health status of coniferous forests (Table 1).

**Table 1:** Datasets and extracted variables used in this study.

Dataset	Abbreviation	Temporal res. and coverage	Spatial res.	Variables
Normalized difference vegetation index Copernicus [26]	NDVI	10 days, 01.01.2017–December 31, 2020	300 m	Annual Productivity Minimum cover Seasonality
Digital elevation model [33]	DEM	February 11, 2000 to February 21, 2000	30 m	DEM
CORINE Landuse/land cover [49]	LULC	2018	100 m	LULC
Monthly total precipitation (mm) [40]	MTP	30 days, 01.01.2017–31.12.2020	1000 m	Cumulative Minimum Variation of each climate dataset
Mean of the monthly averaged minimum daily air temperature (1/10 °C) [41]	MMT	30 days, 01.01.2017–31.12.2020	1000 m	
Monthly total sunshine duration (h) [42]	MTS	30 days, 01.01.2017–31.12.2020	1000 m	
Monthly drought index (de Martonne index) [43]	dMI	30 days, 01.01.2017–31.12.2020	1000 m	
Coniferous forest damage map [47]		2018 and 2019	10 m	Damaged area
Orthophotos [50]		2018 and 2019	20 cm	Identify and verify Damaged and non-damaged sites

#### 2.2.2 Climate data

Various climate datasets related to vegetation and ecophysiology of plants were obtained from the Climate Data Center (CDC) (https://cdc.dwd.de/portal/) including grids of monthly total precipitation [40], monthly mean of minimum daily air temperature in 2 m height above ground [41], monthly total sunshine duration [42], as well as grids of monthly drought index (de Martonne index = dMI [43,44]). The dMI is calculated based on Equation (1).

(1) $$\left[44\right]:dMI=\frac{P}{T+10}$$

T refers to the temperature in degrees Celsius from temperature grids and P refers to the precipitation in mm from precipitation grids. This index shows the aridity of regional climate zones and provides information on the drought level at a given site [45]. Lower dMI values indicate drought while increased values indicate more water is available for trees. The dMI has been used to describe the extreme drought conditions for summer 2018 in Central Germany [46].

In order to investigate the relationship between DHI and climatic drivers, we calculated the cumulative, minimum, and variation of the climate variables (Table 1).

#### 2.2.3 Coniferous forest data

Geodata on the damage of coniferous forests were obtained from the Hessian state forestry service [47]. Since bark beetle outbreaks following the deep depression Friederike in January 2018, the forestry service regularly monitors coniferous forest health in early summer [47]. Damaged forests are detected based on multispectral Sentinel 2 data and pixel-based change detection using the NDVI [47]. Random Forest [48] was used to generate a spatial analysis mask, describing the exact distribution of coniferous forests in summer 2017, before the winter storm Friederike and the associated bark beetle outbreak. This mask allows to define sharp thresholds for damage detection resulting in complete spatiotemporal patterns of forestry damage [47]. To identify non-damaged areas, the CORINE Land-use and Land cover (LULC) map was used to extract coniferous forests in Hesse [49]. Coniferous forest patches displaying a homogenous green texture based on digital orthophotos (DOP) from the geoportal of Hesse [50] were classified as non-damaged. (Fig. 1C). After that, we applied spatial thinning using “spThin”, and spatial autocorrelation was checked using “acf” R package [51]. We found no spatial autocorrelation in our dataset.

### 2.3 The Hessian Dynamic Habitat Index (DHI_Total_)

NDVI data from 2017 to 2020 were recorded with an interval of 10 days [26], thus three tiles were collected per month resulting in 36 tiles for each year and a total of 144 tiles for the full 4-year dataset. For each year, yearly NDVI composites were used to obtain the three DHI components, i.e., cumulative DHI (DHI_Cum_), minimum DHI (DHI_Min_), and variation DHI (DHI_Var_). Here, the three components of DHI_Total_ were computed over the time period 2017–2020 from the NDVI layers based on Equation (2) [52]:

(2) $${DHI\;}_{Cum}=\sum p_{t}\;{DHI}_{\min}=\min\left(p_{t}\right)\;{DHI}_{Var}=\frac{\sigma \left(p_{t}\right)}{\mu \left(p_{t}\right)\;}\;t=1..n$$

where p represents vegetation productivity at different periods (t) during a year [25,53]. DHI_Cum_ refers to the cumulative productivity values for all time periods over a year, DHI_Min_ refers to the minimum productivity value within a year, and DHI_Var_ refers to the seasonality of the productivity calculated based on the coefficient of variation using the standard deviation and the mean ($\sigma \left(p_{t}\right)/\;\mu \left(p_{t}\right)$) [25,53]. In addition, the long-term mean of each indicator was calculated as the median over the full four-year period. Further details on DHI calculation are described by Refs. [21,25,28,54].

To reveal the extent to which the three DHI components complement each other [55], we performed Spearman correlation analyses between DHI_Cum_, DHI_Min_, and DHI_Var_. In addition, we tested the correlation of the DHI components with the climatic variables under study.

### 2.4 Yearly comparison

One-way analysis of variance (ANOVA) was performed to test differences in DHI components across damaged and non-damaged coniferous forests within each of the four years using the “car” package [51]. We met the assumptions of one-way ANOVA using the Shapiro-Wilk test, Q-Q plot of residuals, and Bartlett's Test [56]. To test for immediate or time-delayed effects of the severe drought in 2018 and for possible recovery, we calculated the change of the DHI components between each combination of years.

We performed Theil–Sen's test and Ordinary Least Squares (OLS) with a moving widow [57,58] to investigate the overall trends of the three DHI components. Theil–Sen's test is a non-parametric and robust method concerning missing values and non-normal distributions [59]. To assess the effect of drought on the observed trends, we first classified cumulative drought into four zones including arid, semi-arid, semi-humid, and humid and identified areas with high aridity over time. These classes which indicated the aridity of regional climate zones came from equal intervals of cumulative drought maps. To figure out which cells had a considerable trend across the time we applied a 20% threshold on the DHIs components on the entire distribution of trend values [54,60]. Finally, we used the positive-to-negative trend ratio to understand how health status was affected by which arid zone.

### 2.5 Environmental variables across health status

To distinguish which variables were related to the health status, and to gain a better understanding of the trend analysis, we added further environmental variables such as cumulative, minimum, and variation precipitation, temperature, sunshine duration, and monthly drought index (Table 1) to our analysis. Strongly intercorrelated variables (ǀRǀ >0.8) were removed [61]. We used logistic regression (LR) as a subtype of generalized linear models (GLM) and chose binomial distribution [62] as the response data are binary (damaged, non-damaged) using the “glm” function in R statistical software [51]. The model was built based on Equation (3).

(3) $$p\left(X\right)\frac{e^{\beta 0+\beta 1X1+\beta 2X2+\ldots +\beta \text{pXp}}}{\left({1+e}^{\beta 0+\beta 1X1+\beta 2X2+\ldots +\beta \text{pXp}}\right)}$$

*p*: the probability of occurrence of an event, β_0_: model constant, β_i_: regression coefficients, and X_i_: environmental variables [63]. To assess how well the LR model fits the data, McFadden's R2 using “pR2” function from the “pscl” package was employed. Values close to 0 indicate low goodness of-fit, and values over 0.40 indicate a very good model fit [64]. The importance of each environmental variable using the “varImp” function from the “caret” package was computed. To minimize effects of multicollinearity, variables with variance inflation factor (VIF) > 5 were removed [65].

**Fig. 2:**
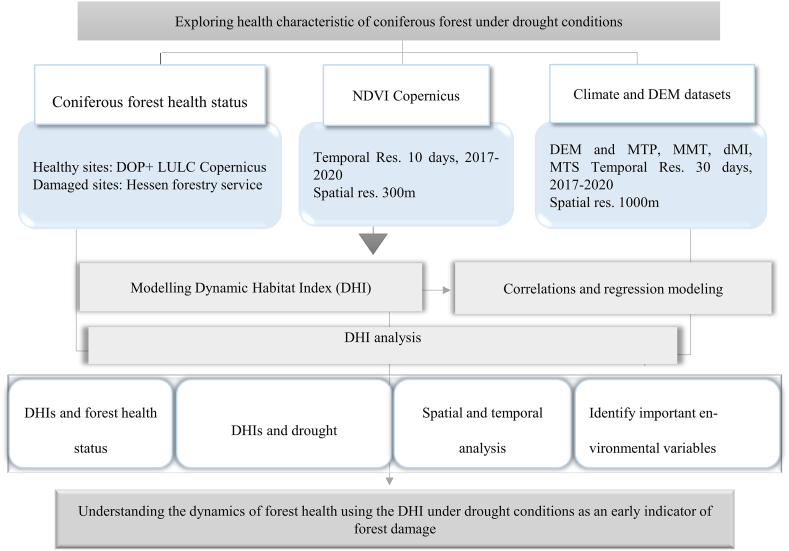
Methodological steps of the inputs, analysis, and outputs. DOP: Digital orthophotos, DEM: digital elevation model, MTP: monthly total precipitation, MMT: mean of the monthly averaged minimum daily air temperature, dMI: Monthly drought index (de Martonne index), and MTS: monthly total sunshine duration, DHI: Dynamic habitat index, and LULC: Land Use Land Cover.

All statistical and geospatial analyses were performed in R statistical software ver. 4 [66] and QGIS ver. 3.22 LTR Białowieża and gdal wrap [67]. The general framework of our study is summarized in Fig. 2.

## 3 Results

### 3.1 The Hessian Dynamic Habitat Index (DHI_Total_)

**Fig. 3:**
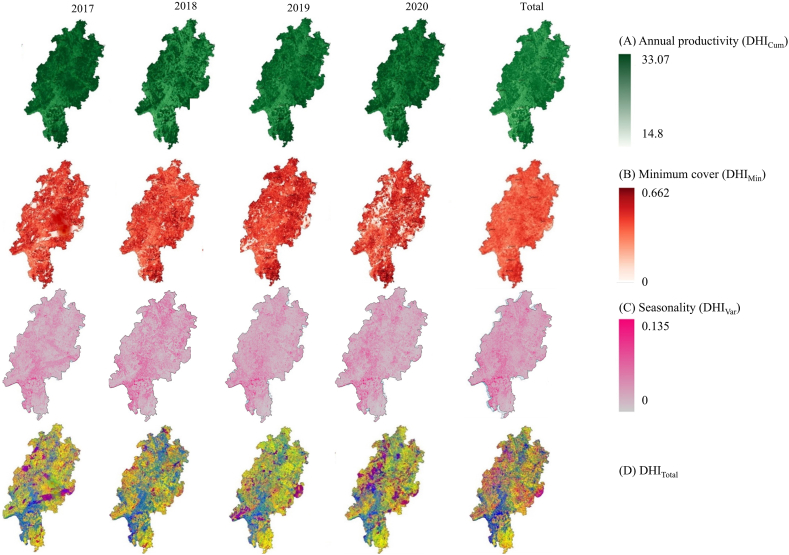
Maps of each year and for all years combinedof the DHI components (A) annual productivity (DHI_Cum_), (B) minimum cover (DHI_Min_), and (C) seasonality (DHI_Var_), which are calculated based on 10-day NDVI data per year, and (D) of the merged components (DHI_Total_) over the 4 years of observations from January 01, 2017 to December 31, 2020.

The three components of the DHI_Total_ varied across time and space (Fig. 3A, B, C) as did the DHI_Total_ (Fig. 3D). DHI_Cum_ (annual productivity) dropped in 2018 (Fig. 3A), while DHI_Min_ (minimum coverage) clearly decreased in 2020 (Fig. 3B). DHI_Var_ (seasonality) in 2018 is different from all other years (Fig. 3C). DHI_Total_ captured the variability in the vegetative productivity patterns during 2017–2020, and the contribution of each DHI component to DHI_Total_ varied between the years (Fig. 3D). For example, while some regions were characterized constantly by high seasonality, low annual productivity and low minimum cover (blueish colours), the location of areas with moderate seasonality, high annual productivity and low minimum cover (reddish purple) varied (Fig. 3D).

**Fig. 4:**
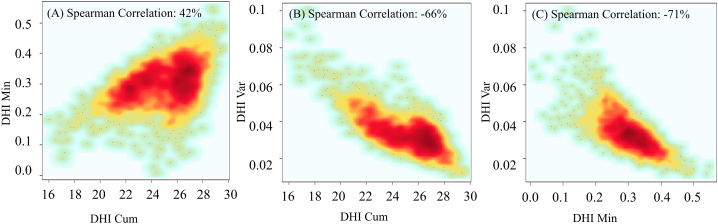
Correlations among the three DHI_Total_ components derived from NDVI. The three DHI_Total_ components were moderate to strongly correlated with each other. DHI_Cum_: Annual Productivity, DHI_Min_: Minimum cover, and DHI_Var_: Seasonality. The color range is based on the density of points. Dense points of each scatter plot were shown with red color and by decreasing points density it changed to orange, yellow, and then green. (For interpretation of the references to color in this figure legend, the reader is referred to the Web version of this article.)

Three DHI_Total_ components were moderately to strongly correlated with each other (Fig. 4). There was a positive correlation between annual productivity and minimum cover values (R^2^ = 0.42). Seasonality was negatively correlated to minimum cover and annual productivity (R^2^ = −0.71 and −0.66, respectively) (Fig. 4B and C). This indicated that the highest values of DHI_Var_ occurred at the lowest DHI_Min_ and DHI_Cum_. The DHI components correlated moderately positively with precipitation, drought, and temperature variables (See Table S1 and Fig. S3).

### 3.2 Yearly comparison between non-damaged and damaged coniferous forests

**Fig. 5:**
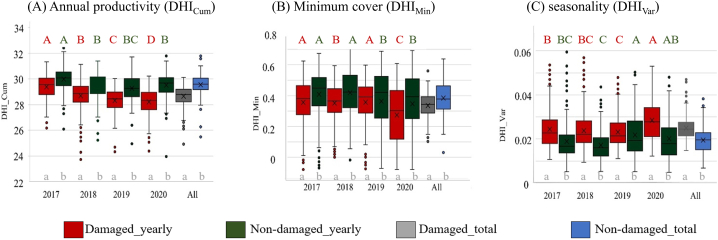
Comparison of damaged and non-damaged coniferous forests for each DHI component for each year (A, B, and C). The capital letters indicate significant differences related to the non-damaged (green letters) and damaged sites (red letters) between years. The lowercase letters in grey indicate significant differences regarding the health status within each year using the Tukey post hoc test (*p*-value<0.05). For example, grey lowercase letters in 2017 mean that there is a significant difference related to the non-damaged and damaged sites. (For interpretation of the references to color in this figure legend, the reader is referred to the Web version of this article.)

Annual productivity and minimum cover were significantly higher for non-damaged sites compared to damaged sites throughout the years (Fig. 5A and B). The seasonality component was constantly significantly lower (Fig. 5C) meaning that in non-damaged sites less variation (seasonality) occurred.

**Fig. 6:**
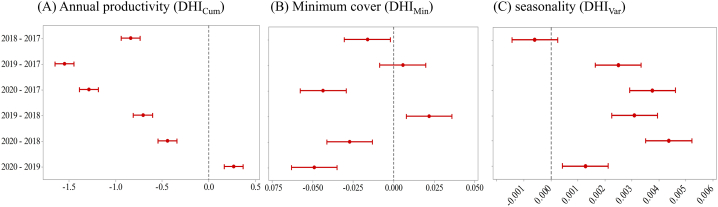
Visualizations of the drought effects across damaged sites. The plots show Tukey's simultaneous tests for differences in DHIs means within years. The confidence intervals display the likely ranges for all of the differences in the means. If an interval does not intersect the zero line, the corresponding means are significantly different (*p*-value<0.05).

When analysing the changes of the DHI components in damaged sites between the years (Fig. 6A), annual productivity first decreased with the most negative changes being observed between 2017 (pre-drought) and 2019 but showed partial recovery in 2020 where it reached its maximum. In contrast, minimum coverage significant decreased in 2020 (Fig. 6B). So, while annual productivity and minimum cover were moderately positively correlated for the whole time span (Fig. 4), yearly comparison showed different and partly opposing trends. The third DHI component seasonality first decreased, but then continuously increased since 2019 (Fig. 6C). Thus, annual productivity in damaged sites was mostly negative, but showed a positive trend and became positive in 2020, while the seasonality increased across all the years apart from a decrease due the 2018 drought event. Minimum cover increased towards 2019 but was negative otherwise. So, all DHI components differed significantly between non-damaged and damaged sites, and they did so even before the drought event 2018 (Fig. 5, Fig. 6). Compared to 2017, damaged sites showed different characteristics after the drought for all DHI components. While annual productivity showed a partial recovery, minimum cover rather decreased and seasonality rather increased (Fig. 6 and Fig. S1).

### 3.3 Spatial and temporal dimension of coniferous forest health status

**Fig. 7:**
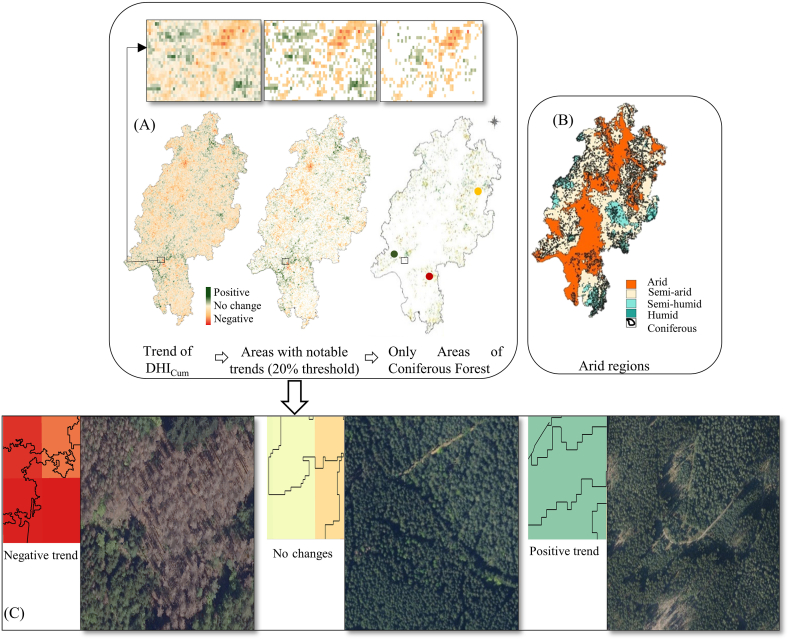
Results of spatial analysis to capture the effects of drought on the DHI's trend (A) Example of DHI_Cum_ trend analysis spanning from 2017 until 2020 (See Fig. S2 for more maps) for coniferous forests. Small boxes above are zoomed in the area to see more details. (B) The accumulated drought map based on the de Martonne index (dMI) ranging from “low accumulated drought” (dark green) to “high accumulated drought” (orange) overlaid with (in black) a coniferous forest map. (C) Examples of coniferous forests showing negative (red), indifferent (yellow) or positive (blue) trends over four years. (For interpretation of the references to color in this figure legend, the reader is referred to the Web version of this article.)

We explored the spatial dimension of DHI changes of coniferous forest from 2017 to 2020 (Fig. 7A) and identified a set these changes into relation with the aridity zones after de Martonne index (Fig. 7B). We then explored the negative or positive trends of the DHI components for different aridity zones based on the dMI (Fig. 7C and Table 2).

**Table 2:** The ratio of the positive trend to the negative trend of the area in each arid zone for each DHI component across health status. Sparklines refer to the ratios across arid zone for each DHI component.

The ratio of positive to negative trend of the area under aridity zones after de Martonne index (dMI).						
arid zones		Arid	Semi-arid	Semi-humid	humid	Sparkline
DHI Components						
Total	DHI_Cum_	2.08	1.39	1.65	2.65	
	DHI_Var_	0.30	0.17	0.04	0.02	
	DHI_Min_	0.89	1.21	2.59	2.72	
Damaged	DHI_Cum_	0,16	0.26	0,33	0,51	
	DHI_Var_	0.55	0.43	0.09	0.04	
	DHI_Min_	0.70	0.86	2.01	3.00	
Non-damaged	DHI_Cum_	1.95	1.20	1.43	2.33	
	DHI_Var_	0.29	0.14	0.04	0.02	
	DHI_Min_	0.90	1.29	2.69	2.70	

The area of coniferous forest for each aridity zone equalled 42,284 ha for arid zone (16% of the total coniferous forest area), 165,542 ha for semi-arid zone (62%), 55,108 ha for semi-humid zone1 (21%), and 3243 ha for humid zone (1%). Most positive trends of the DHI components were found at the rather humid end of the dMI aridity gradient (Table 2). For example, the most positive trend of DHI_Cum_ considering all years (2.65, Table 2) was observed when the drought effect decreased (semi-humid zone), while the least positive trend (1.39) was observed when drought increased (semi-arid zone, Table 2). Coniferous forests rarely occurred in areas which were characterized by strong drought effect (arid zone), and this could explain the positive trend in this zone (Fig. 7C). The highest coniferous coverage occurred in the semi-arid zone. The overall trends were similar for non-damaged and damaged forest (Table 2). However, in damaged sites the positive-to-negative ratios of DHI_Cum_ were lower, but DHI_Var_ was higher compared to non-damaged sites (Table 2). DHI_Min_ was generally lower in damaged forest apart from rather humid zone (Table 2).

**Fig. 8:**
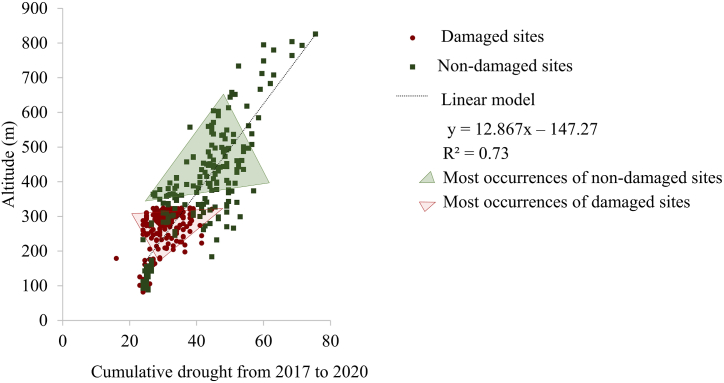
Linear regression between altitude and cumulative drought index (dMI) from 2017 to 2020. Non-damaged sites are displayed in green, and damaged sites are in red. Lower values of cumulative drought indicate stronger drought effects. (For interpretation of the references to color in this figure legend, the reader is referred to the Web version of this article.)

Analysis revealed that most damages of the coniferous forests did not occur in the areas where maximum accumulated drought was located, but rather in the semi-arid zone, where most of the coniferous forest occurred (Table 2). Consequently, most damaged coniferous forests were observed at altitudes lower than 350 m with pronounced, but not maximum cumulative drought. Most of the non-damaged forests occurred at higher altitudes of 350 and lower cumulative drought (semi-humid zone, and y = 12.867x – 147.27 with R^2^ = 0.73) (Fig. 8).

### 3.4 Environmental factors affecting health status

**Fig. 9:**
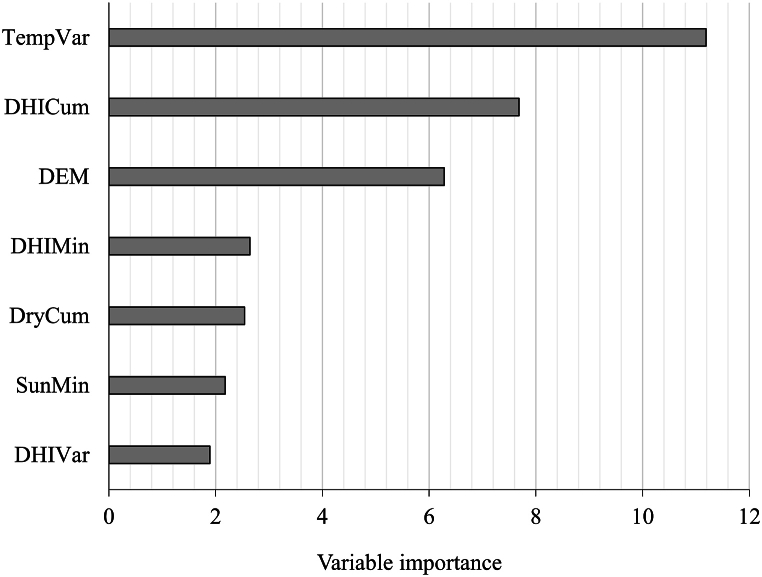
The importance of each predictor variable in the model. Higher values indicate more importance. DHI_Cum_: DHI Annual Productivity. DHI_Var_: DHI Seasonality. DHI_Min_: DHI minimum vegetation cover. Temp_Var_: Variation in the monthly averaged minimum daily air temperature Sun_Min_: Minimum monthly sunshine duration. Dry_Cum_: Cumulative monthly drought index. DEM: Digital elevation model.

Finally, we investigated which environmental factors and which DHI components explained the coniferous health status (Table 3). Out of initially 16 predictors 9 (mainly climatic variables) were removed due to high correlation >0.8 and high collinearity >5, and 7 remained in the final model. Among them were all DHI components (Table 3). Logistic regression analysis revealed that the most important factors explaining the health status were the variation in temperature, annual productivity (DHI_Cum_), altitude, minimum coverage (DHI_Min_), cumulative drought, and minimum sunshine duration (Table 3, Fig. 9). Seasonality (DHI_Var_) was among the most important predictors (Fig. 9), but it was not significant (Table 3). McFadden's R^2^ value of 0.44 indicated that the GLM model with a binomial family fit the environmental data very well [68], and the results of variable importance matched up with the *p*-values from the model (Table 3 and Fig. 9). Thus, in addition to environmental parameters, DHI components were among the most important predictors of the distinction between degraded and non-degraded coniferous.

**Table 3:** Shows the Logistic regression model coefficients and *p*-values. DHI_Cum_: DHI Annual Productivity. DHI_Var_: DHI Seasonality. DHI_Min_: DHI minimum vegetation cover. Temp_Var_: Variation in the monthly averaged minimum daily air temperature Sun_Min_: Minimum monthly sunshine duration. Dry_Cum_: Cumulative monthly drought index. DEM: Digital elevation model.

Coefficients:	Estimate	Std. Error	z value	Pr(>|z|)	Significant^a^
(Intercept)	−54.20	5.33	−10.16	<0.0000002	***
DHI_Cum_	1.27	0.16	7.68	1.55E-14	***
DHI_Min_	−5.59	2.11	−2.64	0.00821	**
DHI_Var_	−49.42	26.13	−1.89	0.05856	.
Sun_Min_	−0.047	0.021	−2.17	0.02931	*
Temp_Var_	0.007	0.0006	11.18	<0.000002	***
Dry_Cum_	0.044	0.017	2.54	0.01105	*
DEM	−0.007	0.001	−6.28	3.32E-10	***

a Significant codes: 0 ‘***’ 0.001 ‘**’ 0.01 ‘*’ 0.05 ‘.’ 0.1 ‘’ 1.

## 4 Discussion

In this study we show how vegetation information derived from satellite time series in the form of Dynamic Habitat Indices (DHI) can be used as indicators and diagnostic tools to assess forest damages caused by drought. All three components of the DHI (annual productivity, minimum cover, and seasonality) were able to capture the response of the coniferous forest to drought, but overall temporal trends differed. We highlight that the drought effect is clearly altitude dependent, but that DHI components are important in addition to climatic variables to distinguish non-damaged from damaged sites.

### 4.1 The potential of the Dynamic Habitat Index to capture the effect of drought on coniferous forest health

DHI was developed as an integrated remote sensing metric to track dynamics of vegetative productivity [53], and to relate these dynamics to biodiversity patterns [69]. As it is based on spaceborne NDVI time series data, it informs on the dynamics of vegetation greenness [53]. The 2018 drought event had a strong negative impact on forest health [46], including coniferous species such as Spruce (*Picea abies*) and Scots pine (*Pinus sylvestris*) [1,70].We found that the DHI based on dense NDVI time series data proved to be a promising diagnostic tool to capture the dynamic changes and drought assessment [71] including forest dieback in the health status of central European coniferous forests as a consequence of the severe drought events.

While surrogates of single components of the DHI such as seasonality [72] or vegetation productivity [73], or minimum NDVI [74] have been used to assess effects of droughts on vegetation, their combination as DHI as rarely used although they combination allows new insights. In this study, each of three DHI components provided useful information to assess forest health status. Coniferous forests generally have high vegetation productivity [75], making both the DHI_Cum_ and DHI_Min_ suitable health indicators for these ecosystems. For example, coniferous forests with high minimum vegetation cover (DHI_Min_) remained healthier in 2018. The lowest values and the largest value ranges of DHI_Min_ occurred in 2020 after two years of drought stress, which could indicate a partial or complete canopy dieback [70] together with a legacy effect [76]. Importantly, not only damaged but also non-damaged sites suffered after two years of drought events. At the same time, partial recovery after the drought year 2018, e.g., related to minimum cover, also occurred, so both positive and negative could be mapped using DHI. However, such increases in NDVI might at least partly be the result of herbaceous vegetation resprouting after partial or complete forest die-off [77], so the forest type might have changed. An indicator for strong drought effects might be the altered vegetation (see Ref. [73]). Here, non-damaged sites showed the least amount of variation (DHI_Var_) compared to the damaged sites. The marked differences for all DHI components value between non-damaged and damaged sites underpin their potential as warning indicators of forest degradation.

### 4.2 Advantages of remote sensing metrics based on times series data

Multiple-year and extended droughts have additive effects on the response of trees in terms of e.g. leaf area [78] and vegetation productivity [79]. So, analysis of temporal changes using time series provides more detailed information about the effects of global change events such as severe droughts on vegetation health compared to a single NDVI tile [80]. We demonstrated that each of the three DHIs components can be useful in terms of drought assessment and showed that Hessian coniferous forests did not fully recover to pre-drought conditions yet. Recovery time as one important component of ecosystem stability is particularly relevant if the frequency and intensities of droughts increase [81]. Ecosystems with longer drought recovery times are more likely to experience a new drought event before fully recovering which leads to increased plant mortality and a potential transition to a new state [82]. These legacy effects are often characterized by ecological responses to water availability and temperature [83]. Therefore, extended droughts affect the recovery time associated with canopy mortality and may push forests beyond a tipping point [46] and ultimately lead to changes in LULC [84]. Our results showed that the time series of NDVIs expressed as DHI indices are helpful for recognizing where damages are considerable and can thus be used as a potential early indicator of forest damage. For example, March 2018 was the start of the drought period that never showed complete recovery by the end of 2020. Similar to our results [46], reported that mean precipitation in April–August 2018 for the growing season was less than half of the normal amount (450–500 mm) in Bavaria in central European forests, and in our study the de Martonne aridity index showed a summer with arid to semi-arid climate (Fig. S. 3 and Fig. S. 4). Decreasing NDVI has been linked to tipping points of *P. sylvestris* forests in Southwest Germany, where the coniferous forest is changing towards a broadleaf forest [16]. Thus, DHI analysis including spatial trends could be used to characterize forest die-off and subsequent recovery. So, changes in DHI might serve as early indicators of forest damage and could be used together with remotely sensed data on tree species identity to explore if there shift towards alternative states such as broadleaf forests or open ecosystems with distinct DHI signatures.

### 4.3 Environmental factors together with the DHIs explain coniferous health status

Severe droughts and heatwaves cause forest die-off in the Antropocene [76,85] and mortality of coniferous tree species is increasing [2,7,9,70,86,87]. Here, in addition to environmental parameters, DHI components were among most important predictors to distinguish non-damaged from damaged coniferous forests, which emphasizes their usefulness in assessing forest drought. An advantage of DHIs derived from satellite data collections (e.g. Ref. [26]) over other climatic datasets (such as precipitation, drought and, temperature) could be that the latter are derived from interpolation techniques and can suffer from potential biases [25]. Nevertheless, variation in temperature was the most important factor to predict health status in our study. Indeed, tree mortality in Europe is triggered by global-change-type drought [88]. Temperature stress and drought could limit the capability of plants to refill cavitated xylem to support metabolism and therefore disable recoverability from drought [82]. It also has a negative impact on wood formation of coniferous forest species [89]. Such drought-affected growth processes are species-specific, show seasonal dynamics and might be altitude dependant [89]. Altitude is also an important factor concerning the magnitude of potential post-drought legacy effects [90]. We found that the drought was particularly severe (cumulative dMI<28) at lower altitude where the area of coniferous forests is relatively small. For coniferous forests, less drought effects occurred at higher altitudes with higher precipitation and lower temperature, which indicated more suitable habitat conditions. In the lower altitudes, the magnitude of the damages to the coniferous on the DHI components increased as precipitation decreased, and coniferous forests were not able to recover in these very dry regions. Thus, the patterns of coniferous forest damage generally followed the altitudinal patterns of drought (Fig. 9). This highlights the importance of considering both the meteorological and other environmental condition particularly to identify cumulative drought effects of a series of dry and vulnerability of coniferous to drought due to the legacy effect possibly persists for a few years after a drought event [76], and DHIs based on dense time series data could play an important role in analysing such legacy effects.

### 4.4 Future directions

Our findings clearly show that the use of DHIs is a promising and straightforward approach to monitor the health status of coniferous forests at larger spatial scales that could contribute additional spatio-temporal information about global change effects such as drought. Previous studies on DHIs were performed at a 1 km spatial resolution on a global scale [55]. Latest DHI developments emphasized the combination of the DHIs, climate, and human-related variables in modelling approaches related to the abundance patterns due to high explanatory power [69]. While we were interested in exploiting the globally available NDVI from the Copernicus program [26,91] whose spatial resolution corresponds to earlier DHI studies [21,25] as well as to the climate data set used here, future studies should explore the potential of high to very high spatial resolution NDVI products based on for example PlanetScope, Sentinel-2 or Landsat data [35]. Further, combination of DHIs with ecophysiological measurements [14] and sensory networks [92] together with high-resolution bioclimatic data sets [93] and synthetic aperture radar (SAR)-based maps on soil moisture [94] and forest drought [95] that potentially identify tree damage even before visible evidence [96]could create new insights into forest-die off due to drought events. While the binary forest damage mask based on freely available Sentinel-2 and a random forest model [47] could be further improved using public aerial imagery and methods of deep learning [97], composite remote sensing indices such as the DHI can be computed at high spatial resolution [98], and could provide a continuous metric to assess forest damage, e.g. to map decreasing annual productivity or stagnation at low level after disturbance. Adding structural data and canopy height information from LiDAR data [99] supported by automated field measurements for calibration [100] would be the next step to improve the identification of tipping points and regime shifts to other types of (forest) ecosystems. A long-term networked monitoring system with relatively high-frequency data is needed to understand the underlying forest dynamics (see Ref. [101]). Depending on the availability of the satellite data and acquisitions date, there are numerous existing images with coarser spatial resolutions at the global scale or finer resolutions at the regional or local scale. Dense NDVI times series can be derived from publicly available Landsat and Sentinel-2 imagery at 30 m [102] or even 10 m resolution [15], and vegetation information can be further scaled down to 3 m resolution using PlanetScope imagery [103]. In addition to NDVI-based information, indices that also include the shortwave infrared spectral bands and that are sensitive to vegetation moisture content can be derived from Landsat imagery and bear high potential to assess climate change effects [104]. So, if the annual DHI profiles of healthy and damaged forests are known and calibrated against ecophysiological field data and if the effects of abiotic factors on DHI profiles are explored (e.g. Ref. [27]) and computational challenges are met, then such time-series based information could contribute to an automated warning system, e.g., by indicating deviations from healthy forest DHI profiles in a spatially explicit way.

## 5 Conclusion

Progress in earth technology facilitates its application in ecosystem studies, e.g., to quantify landscape dynamics and capture changes due to extreme droughts, particularly when using time series of remotely sensed and freely accessible data. The DHI based on the multiyear NDVI time series clearly illustrated the strong effect of the drought year 2018 on coniferous forests, so it could support monitoring health status in central European coniferous forest ecosystems or further LULC types. Comparing the values of three DHI components across non-damaged and damaged sites could help to develop early warning indicators of ecosystem degradation and changes in ecosystem functioning. This study also highlighted the importance of considering meteorological and environmental conditions to interpret the remote sensing-based assessment of ecosystem condition. Therefore, we suggest testing this approach across different LULCs types or to assess potential additive effects of multiple extreme events. Future avenues include the use of very high-resolution optical time series data as well as the integration of other data types such as LiDAR to map changes in vegetation structure, or SAR time series data to explore, e.g., changes in vegetation structure and soil moisture especially in areas where the use of optical data is limited.

## Availability of data

Data available on request from the authors.

## CRediT authorship contribution statement

**Mojdeh Safaei:** Writing – review & editing, Writing – original draft, Visualization, Validation, Methodology, Investigation, Formal analysis, Data curation, Conceptualization. **Till Kleinebecker:** Writing – review & editing, Supervision, Methodology, Conceptualization. **Manuel Weis:** Writing – review & editing, Methodology, Data curation. **André Große-Stoltenberg:** Writing – review & editing, Validation, Supervision, Resources, Methodology, Data curation, Conceptualization.

## Declaration of competing interest

The authors declare that they have no known competing financial interests or personal relationships that could have appeared to influence the work reported in this paper.
