# Supplementary material for: Tracking effects of extreme drought on coniferous forests from space using dynamic habitat indices
Source: Heliyon. 2024 Mar 20;10(7):e27864. doi: 10.1016/j.heliyon.2024.e27864 (PMC10981029; doi:10.1016/j.heliyon.2024.e27864)
Supplement: Multimedia component 1 [file mmc1.docx]

Tracking effects of extreme drought on coniferous forests from space using Dynamic Habitat Indices

Mojdeh Safaei^a*^ Till Kleinebecker^a, b^ Manuel Weis^c^ André Große-Stoltenberg^a, b^

a Division of Landscape Ecology and Landscape Planning, Institute of Landscape Ecology and Resource Management, IFZ Research Centre for Biosystems, Land Use and Nutrition, Justus Liebig University Giessen, Heinrich-Buff Ring 26-32, 35392 Giessen, Germany

b Center for International Development and Environmental Research (ZEU), Senckenbergstrasse 3, 35390 Giessen, Germany

c Hessian Agency for Nature Conservation, Environment and Geology (HLNUG) Rheingaustraße 186, 65203 Wiesbaden, Germany

Corresponding author: [mojdeh.safaei@umwelt.uni-giessen.de](https://webmail.thm.de/rc/?_task=mail&_caps=pdf%3D1%2Cflash%3D0%2Ctiff%3D0%2Cwebp%3D1&_uid=1210&_mbox=INBOX&_framed=1&_action=preview#NOP), Tel : 0641 / 99-37177 and andre.grosse-stoltenberg[@umwelt.uni-giessen.de](https://webmail.thm.de/rc/?_task=mail&_caps=pdf%3D1%2Cflash%3D0%2Ctiff%3D0%2Cwebp%3D1&_uid=1210&_mbox=INBOX&_framed=1&_action=preview#NOP), Tel : 0641 / 99-37176

MS ORCiD: <https://orcid.org/0000-0002-6509-8307>

TK ORCiD: <https://orcid.org/0000-0003-1121-2861>

AGS ORCiD: <https://orcid.org/0000-0001-6075-5497>

# Supplementary material

## Table S1

Correlation between the DHI component and environmental components is presented in Table S1 (*p*-value<0.001). Annual productivity, precipitation and drought variables correlated moderately and positively. In the case of the drought variable derived from dMI, higher values indicate humid environmental conditions. A moderate and positive correlation between the annual productivity and minimum as well as temperature. Relatively weak relationships were obtained for sunshine components and no correlation with DEM data was found. In the case of DHI_Var_, the highest correlation with the precipitation cumulative and variation were only −0.35 and -0.34, and correlation with cumulative and variation of the drought were -0.33 and -0.31. DHI_Min_ was (at maximum) weakly correlated with climate variables and DEM data.

Table S1 Correlations between the three DHI_Total_ components derived from NDVI and the environmental variables (p-value < 0.001)

| DHI Components  Variables | | Annual productivity | Minimum cover | Seasonality |
| --- | --- | --- | --- | --- |
| Precipitation | Cumulative | 0.54 | 0.10 | -0.35 |
|  | Minimum | 0.38 | 0.01 | -0.21 |
|  | Variation | 0.52 | 0.10 | -0.34 |
| Temperature | Cumulative | -0.31 | 0.05 | 0.15 |
|  | Minimum | -0.38 | -0.01 | 0.26 |
|  | Variation | 0.10 | 0.11 | -0.07 |
| Sunshine duration | Cumulative | 0.01 | 0.07 | -0.08 |
|  | Minimum | 0.19 | 0.11 | -0.17 |
|  | Variation | -0.21 | -0.07 | 0.12 |
| Drought | Cumulative | 0.55 | 0.06 | -0.33 |
|  | Minimum | 0.33 | 0.01 | -0.22 |
|  | Variation | 0.52 | 0.05 | -0.30 |
| DEM | | 0.0 | -0.23 | 0.05 |

## Figure S1


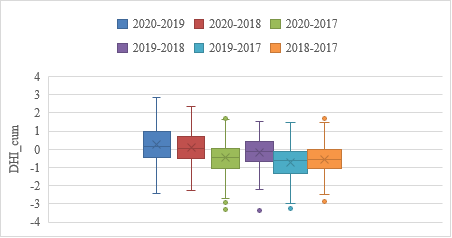

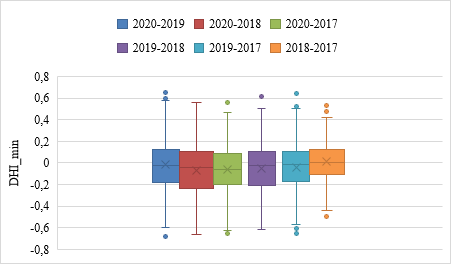

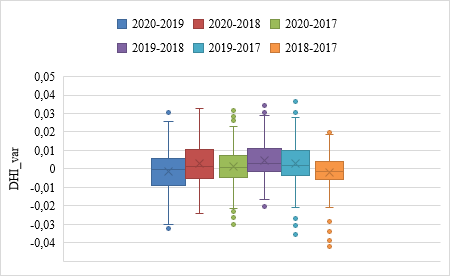


Fig. S. 1. Differences between DHI components across different years in healthy sites.

## Figure S2

(A) Annual production (B) Minimum cover (C) seasonality


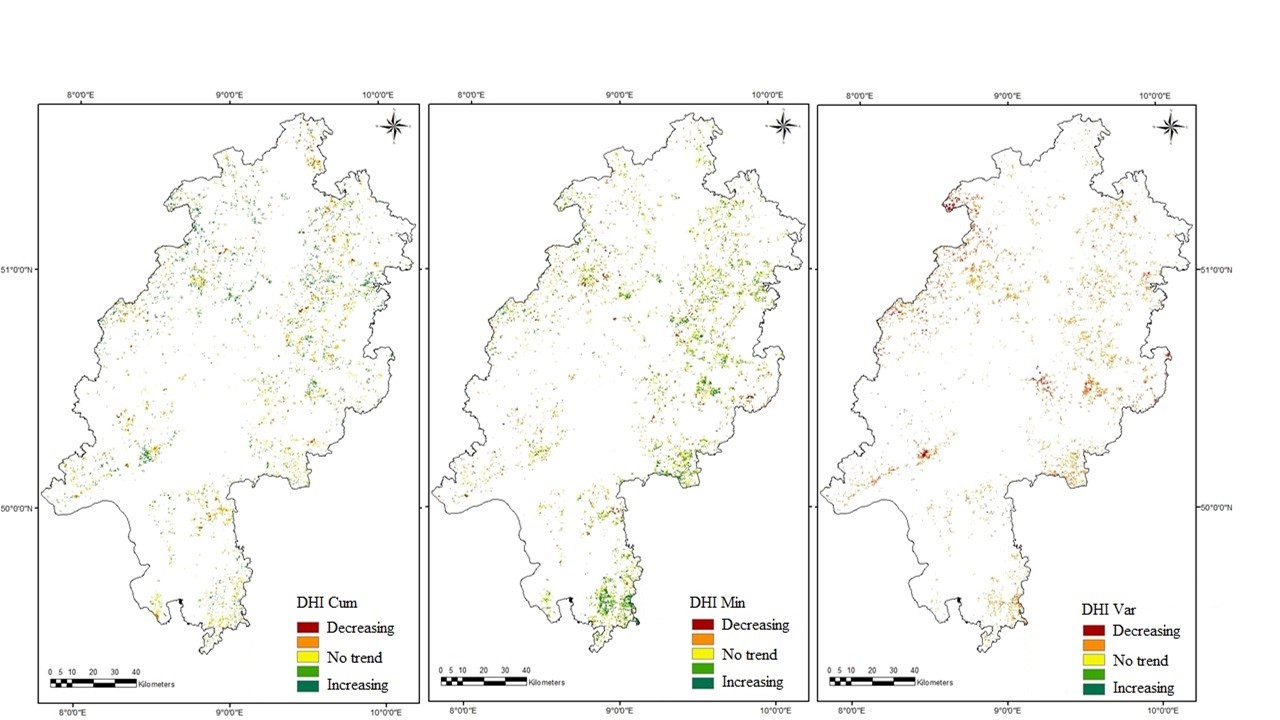


Fig. S2. Results of trend analysis to capture the effects of drought on the DHI components (A) DHI_Cum_ (B) DHI_Min_ and (C) DHI_Var_. Trend analysis was performed using Theil–Sen’s test. A 20% threshold was applied to the DHIs components on the entire distribution of trend values to figure out which cells had a considerable trend spanning over the 2017–2020 period.

## Figure S3


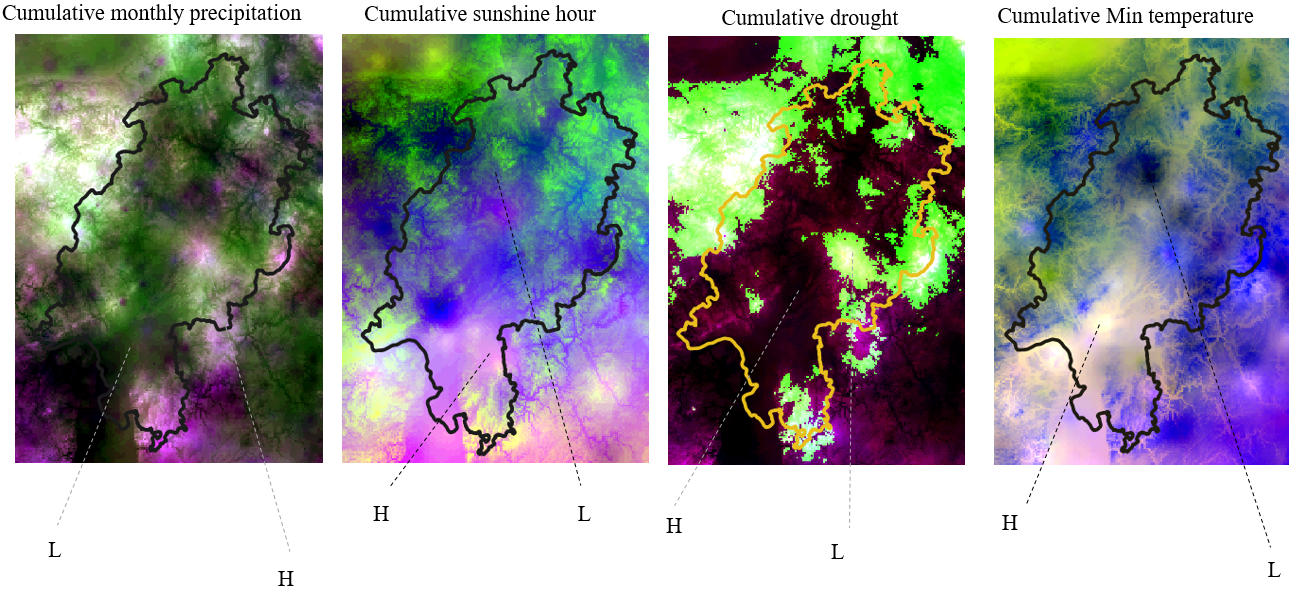


Fig S3. Environmental variable maps. H:Higher values and L: Lower values.

## Figure S4

21.07.2017 01.03.2018
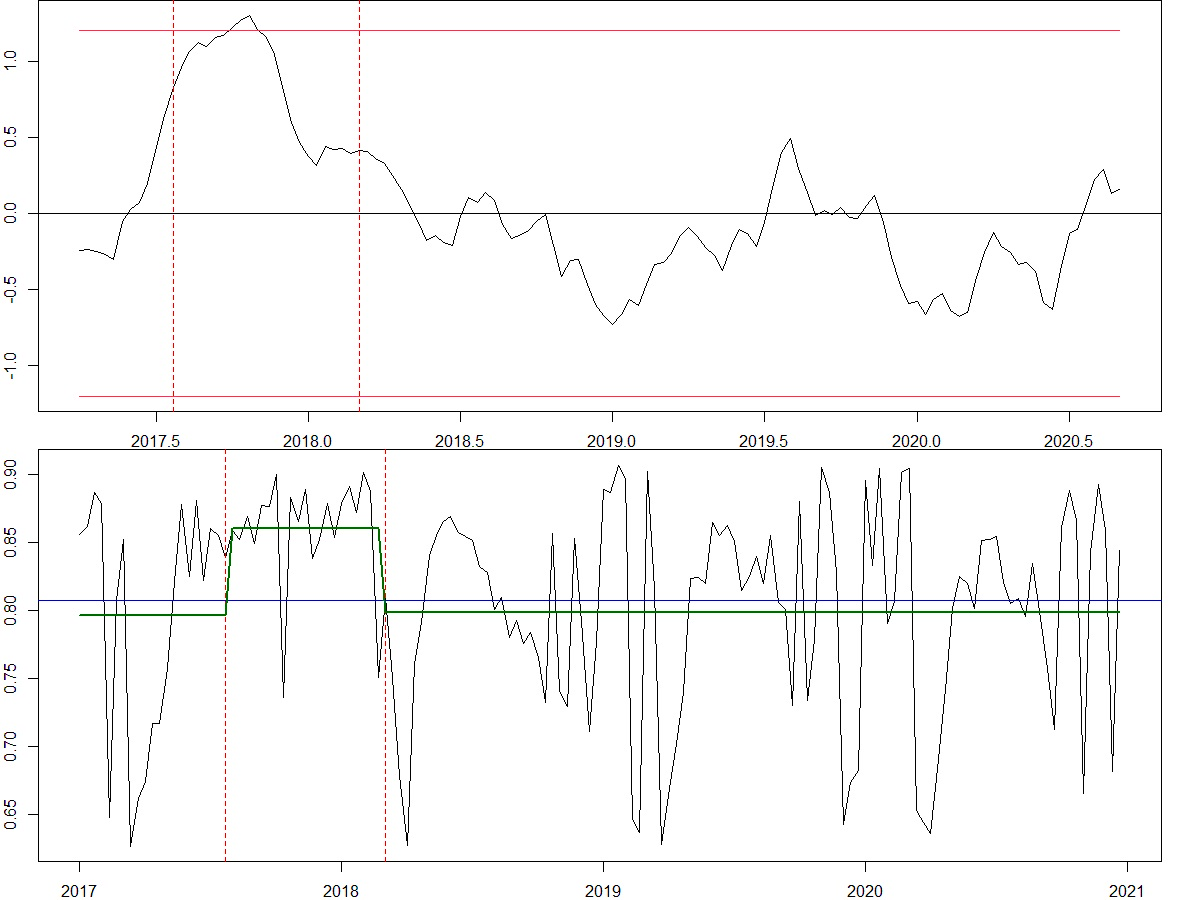


Fig S.4. Time series analysis revealed two important breakpoints. A positive trend started in late July 2017 until the end of February 2018 and then a decreasing trend in NDVI was identified in March 2018. This pattern was partly in line with precipitation trends, where in 2017 the amount of precipitation was markedly higher than in 2018 and a negative breakpoint was identified in February 2018. By the end of August 2018, the amount of precipitation increased (second positive breakpoint) but did not fully recover to the level of 2017.


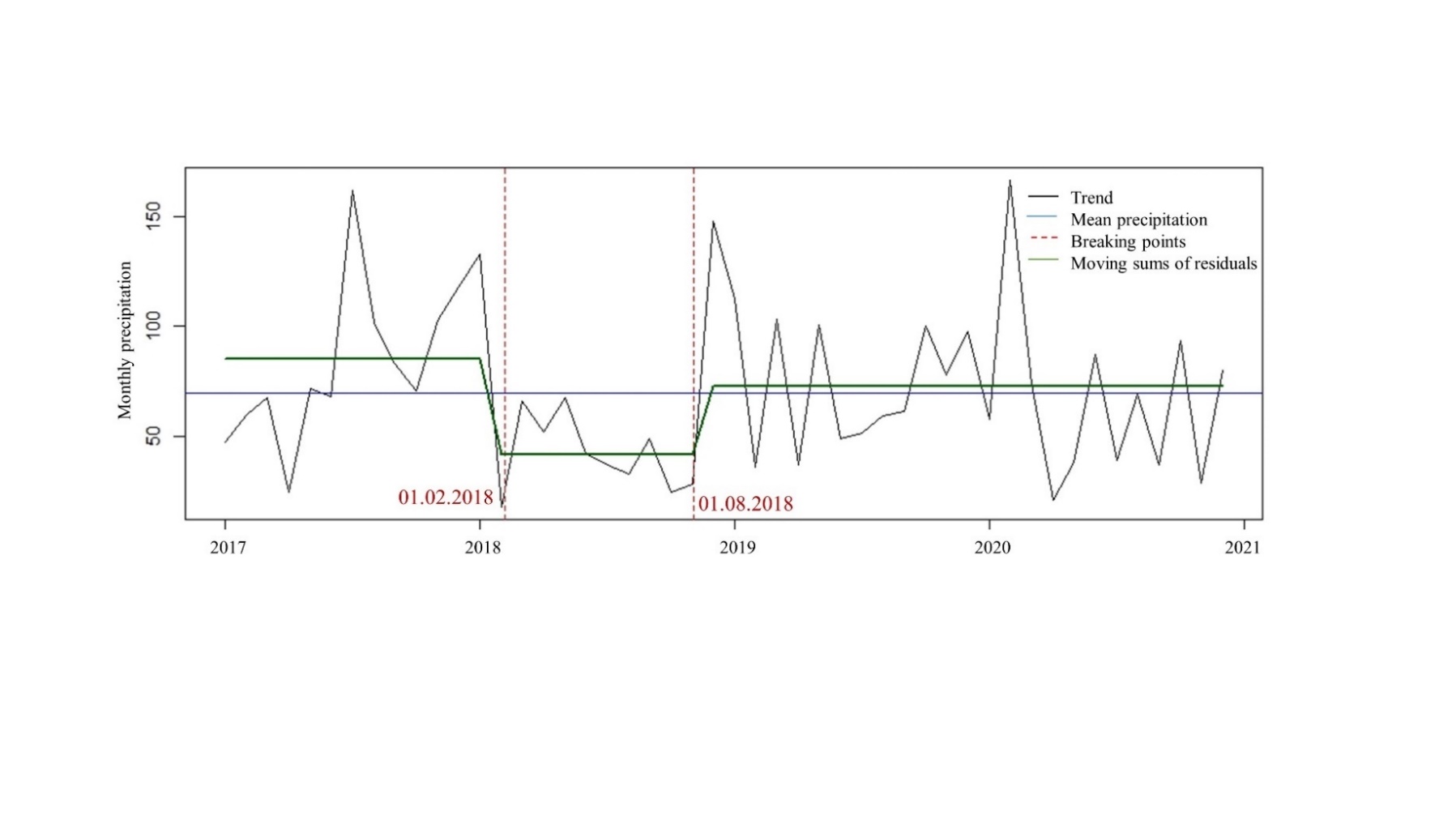
 Monthly precipitation trends: in 2017 the amount of precipitation was higher than in 2018 and a negative breakpoint started from February 2018 to August 2018 and covered the complete growing season in 2018, and by the end of August 2018, the amount of precipitation increased as the second positive breakpoint but failed to match the 2017 trend.
